# Association between Multiple Myeloma and Ulcerative Colitis: A Cross-Sectional Analysis

**DOI:** 10.3390/diseases11020059

**Published:** 2023-04-06

**Authors:** Ayrton Bangolo, Sowmya Sagireddy, Paul Desrochers, Imane Laabidi, Vignesh K. Nagesh, Amer Jarri, Imranjot Sekhon, Youssef Laabidi, Deeksha Muralidhar, Adarshpreet Singh, Paranjyothy R. P. Sanjeeva, Damanpartap S. Sandhu, Saba Salma, Saad A. Khan, Mir I. Ali, Sung H. Kim, Wardah Bajwa, Angela C. Tai, Assma Itani, Kareem Ahmed, Mevlut Ozmen, Bhargav Hirpara, Shruti M. Borse, Simcha Weissman

**Affiliations:** 1 Department of Medicine, Hackensack Meridian Health Palisades Medical Center, North Bergen, NJ 07047, USA; 2 Department of Medicine, University of Washington, Seattle, WA 98195, USA

**Keywords:** multiple myeloma, plasma cell dyscrasia, IBD, ulcerative colitis, cross sectional analysis

## Abstract

Background and Aims: Multiple myeloma (MM) is a plasma cell dyscrasia that is common among patients with autoimmune diseases. However, the association between ulcerative colitis (UC) and multiple myeloma (MM) is yet to be established. We aimed to evaluate the prevalence of MM among patients with UC in the United States. Methods: This cross-sectional cohort analysis used the National Inpatient Sample from 2015–2018 to assess the overall MM prevalence among patients with and without UC, and within specific demographic subgroups. Prevalences were compared using a logistic regression model controlling for sex and age. Results: The crude prevalence of MM among patients with UC (*n* = 1750) compared with patients without UC (*n* = 366,265) was 0.44% vs. 0.37%, respectively. Patients with UC had increased overall odds of having MM (odds ratio (OR), 1.26). Males with UC had higher prevalence of MM (53.7% vs. 46.3%, respectively) than females. Patients with UC and MM were more likely to be African American than White (15.6% vs. 9.2%, respectively). Patients with UC age >64 had a higher prevalence of MM than those aged below 65 (70.9% vs. 29.1%, respectively). Patients with UC who were obese (BMI > 30) had a higher prevalence of MM than those who were non-obese (12.6% vs. 8.3%). Conclusions: Overall, UC appears to be associated with MM. This association can be particularly observed in specific demographic groups, such as obese, African American males, or patients >64 years of age. Thus, a high degree of clinical suspicion for MM is warranted, even with minimal symptomatology, in patients with UC, in particular among elder, obese, and African American males.

## 1. Introduction

Multiple myeloma is a rare malignancy caused by plasma cell proliferation leading to a monoclonal expansion of immunoglobulin in the bone marrow. MM represents approximately 2% of all cancers and about 17% of all hematologic malignancies [1,2,3]. Symptoms of MM are attributed to plasma cell penetration in target organs such as the kidney. The clinical presentation is often subacute [1]. Renal derangements happen in more than half of patients with MM, with hypercalcemia being the most common cause. Other contributing factors can include the use of nonsteroidal anti-inflammatory agents for pain control, amyloid deposits, iodine contrast for imaging, and high levels of serum uric acid [1,2,3].

To make a diagnosis of MM, laboratory exams, and radiologic and pathologic findings are needed. Serum protein electrophoresis can detect the colony of monoclonal antibody (M protein), which is secreted by most MM patients. Immunofixation or mass spectrometry help identify the different components of the M protein, in heavy and light chains (e.g., IgA, IgG, kappa, or lambda light chain). Bence Jones proteins, which are free light chains, can be detected in the urine using previously mentioned techniques; more recently, Bence Jones proteins have been detected in the serum using highly sensitive free light chain tests [1,2,3]. MM has a higher incidence among patients with autoimmune disease [1].

Inflammatory bowel disease (IBD) is a chronic, autoimmune, inflammatory, intestinal condition—primarily affecting patients in their third or fourth decades of life. Ulcerative colitis presentation consists of weeks to months of bouts of bloody bowel movements. Recurring symptoms are observed during treatment, with long periods of compete subsidence of symptoms. It is estimated that up to 67% of patients will have a relapse in the 10 years following diagnosis, with age at diagnosis being a key indicator of relapse [4,5]. Owing to its chronic inflammatory nature, IBD has long been associated with numerous malignant conditions, most notably colorectal cancer [4,5].

In IBD, there is a compromise of the first line of mucosal defense, leading to a systemic response of antigens crossing the intestinal mucosal barrier. As a result, B-lymphocytes and plasma cells are activated, which could explain the causal relationship between MM and IBD [6]. Furthermore, the use of biologics such as tumor necrosis factor-α (TNF-α) inhibitors in IBD can lead to decreased apoptosis of plasma cells, thus setting the stage for the development of MM [7].

Although ulcerative colitis (UC) and multiple myeloma (MM) are reported to coexist, albeit in the form of small case-series, the association between MM and UC is yet to be established, nor has it been systematically evaluated [4,5]. While MM in general has been shown to be most prevalent among elderly, African American males, the corresponding demographic profile among patients with comorbid UC remains illusory.

To fill in the gap in the literature, using a large United States (US) population-based dataset, we aimed to evaluate the prevalence of MM among patients with UC and to determine the strength of association between these two medical conditions.

## 2. Methods

### 2.1. Study Design

The study used the standardized electronic health record information of the National Inpatient Sample (NIS) (https://hcup-us.ahrq.gov/nisoverview.jsp, accessed on 16 March 2021), which is the largest publicly available inpatient database in the United States, containing data on more than seven million hospital stays [8]. The sample is from 2015–2018.

### 2.2. Data Selection

Inclusion Criteria:

The analysis was limited to adults aged at least 18 years between 2015–2018, who had complete information on sex and age. ICD-10 codes corresponding to multiple myeloma (C50) and ulcerative colitis (K51) were used to identify cohorts. The use of electronic health data to identify UC and MM cohorts has been performed in prior studies evaluating the associations between other conditions [9,10].

Exclusion criteria:

We excluded patients younger than 18 years, with an unknown ethnicity, BMI, or sex.

### 2.3. Study Variables

Variables included in our study are age, sex, ethnicity, and BMI.

### 2.4. Statistical Analysis

A logistic regression model that controlled for sex and age was used to compare prevalences. Two-tailed hypothesis tests were used, and a threshold of statistical significance was considered with a *p* value < 0.05. Software STATA 16.1. was used to perform all of the statistical tests.

The NIS dataset is a public-use dataset, for which informed consent is waived.

This study does not require institutional board review as it contains deidentified, publicly available data.

## 3. Results

From a total of 98,355,141 patients hospitalized between 2015–2018, we identified 393,630 patients with UC and 367,415 with MM. The crude prevalence of MM among patients with UC (*n* = 1750) compared with patients without UC (*n* = 366,265) were 0.44% vs. 0.37%, as seen in Table 1. Upon multivariable analysis adjusting for age, sex, ethnicity, and BMI, patients with UC had increased overall odds of having MM (odds ratio (OR), 1.26; 95% CI, 1.04–1.58). The prevalence of MM among UC patients stratified by subgroups is reported in Table 2.

### 3.1. Subgroup Based on Sex

Males with UC had a higher prevalence of MM compared with females with UC (53.7% vs. 46.3%). Significant effect modification was observed in the association between UC and MM across sex subgroups as the ORs for the association between UC and MM were higher in males (OR, 1.36; 95% CI, 1.02–1.87) than in females (OR, 1.14; 95% CI, 1.03–1.32) (*p*  =  0.03).

### 3.2. Subgroup Based on Ethnicity

Regarding ethnic groups, African Americans had a higher prevalence of MM among patients with UC compared with White patients (15.6% vs. 9.2%). Significant effect modification was observed in the association between UC and MM across ethnic subgroups as the ORs for the association between UC and MM were higher in African American patients (OR, 1.31; 95% CI, 1.11–1.63) than in White patients (OR, 1.05; 95% CI, 1.02–1.18) (*p*  =  0.001).

### 3.3. Subgroup Based on Age

Patients with UC aged 65 years or older had a higher prevalence of MM than those aged below 65 (70.9% vs. 29.1%, respectively). Age was a significant effect modifier in the association between UC and MM. Patients with UC aged greater than or equal to 65 years had 2.7 (95% CI, 2.38–2.85) times the odds of MM compared with patients without UC who were under 65 (*p*  < 0.001 for the interaction of MM with age).

### 3.4. Subgroup Based on Body Mass Index (BMI)

Patients with UC who were obese (BMI > 30) had a higher prevalence of MM than those who were non-obese (12.6% vs. 8.3% respectively). Obesity was a significant effect modifier in the association between UC and MM. Patients with UC who were obese had 2.2 (95% CI, 2.17–2.61) times the odds of MM compared with patients without UC who were non-obese (*p*  < 0.001 for the interaction of MM with obesity).

## 4. Discussion

By way of this large, nationally representative cross-sectional analysis, we demonstrated that there is a significant association between MM and UC. Furthermore, the results from specific demographic data revealed that among the UC cohort, African American males, >64 years of age, and obese patients are at higher risk for MM.

The distribution of MM in our cohort is in concordance with the current literature. Studies by Kyle et al. and Bladej et al. revealed that MM is largely a disease of older adults with a median age at diagnosis of 65 to 74 years [11,12]. Several studies in the United States of America (USA) and the United Kingdom have demonstrated that the incidence of MM in African Americans and Black populations is two to three times higher than in White populations [11,13,14,15]. MM is also slightly more frequent in men than in women, and a similar trend is seen with UC [11,16]. The age of onset for many patients with UC is between 15 and 30 years, although IBD can present at any age. The literature suggests a bimodal age distribution for UC with a later peak after the age of 50 that can be attributed, to some degree, to a higher susceptibility of the pathology at an older age, continuous environmental exposure, or higher usage of healthcare resources in the elderly. Autoimmunity also plays a key role in the occurrence of this second peak. This second peak coincides with the higher prevalence of MM among older patients with UC [17,18]. The incidence of UC is lower in Black populations compared with White populations. This is thought to be related to environmental and lifestyle factors, as well as underlying genetic differences [19,20].

These results are especially important in the era of biologics, which are widely used in the management of UC, either to induce or maintain remission [21]. It has been shown in the literature that the use of biologics is associated with the development of MM [22]. Taking that into account, one would advise careful use of biologics in elderly Black patients while managing UC. However, it has been shown that Black patients with multiple myeloma have better survival than White patients when treated equally [23].

Patients with a higher BMI are at increased risk for MM [24,25,26,27]. Although it is unclear whether obesity is associated with an increased risk of developing UC [28], the accumulation of intra-abdominal fat may contribute to mucosal inflammation, thereby affecting the clinical course in patients with established UC [29]. Obese patients with UC had a higher prevalence of MM in our cohort. Additionally, higher cancer mortality can be observed in obese and overweight patients [30]. Sex hormone imbalance, derangements in insulin and insulin-like growth factor levels, and adipose pathways are potential explanations [31]. The rates of cancer related to excess weight are higher in females than males [25]. Further studies will be required to assess how gender and BMI affect the incidence and mortality of MM among patients with UC. MM and obesity have been strongly associated in the literature through multiple meta-analyses [25]. A low suspicion index of MM should be maintained among UC patients that are obese.

Patients with inflammatory bowel disease, particularly Crohn’s disease (CD), have been shown to be at increased risk for extra intestinal malignancies [32]. While medical therapies (immunomodulators and biologics) may reduce the incidence of inflammation-associated cancer, some therapies are associated with increased risk of malignancy, most commonly skin cancers and b-cell lymphomas [33]. The biological pathways involved in hematological malignancies and UC are interlinked, as numerous mediators of inflammation, such as tumor necrosis factor-alpha, nuclear factor kappa-light-chain-enhancer of activated B cells, and interleukins, are shared key components of the pathogenesis of both MM and UC [33,34].

Chronic inflammation similar to what is observed in UC can lead to the stimulation of B lymphocytes, which in turn will promote the expansion of plasma cells in a monoclonal fashion. As a result, monoclonal gammopathy of undetermined significance (MGUS) may arise. Regarding MGUS, being a precursor of MM, one can conclude that there is an association between MM and IBD [34]. However, to the best of our knowledge, no sufficiently powered studies have been carried out to investigate this clinically relevant association.

Several cases of IBD associated with MM have been reported in the literature. Fadem et al. reported a case of plasmacytoma in a patient with UC [35]. In the mid-1960s, Bernstein and Dixon discussed a patient with UC who was diagnosed with MM [36]. Around the same time, Rockl et al. investigated six patients with pyoderma gangrenosum and ulcerative colitis and found that four patients had MM [37]. These associations were thought to be of a chronic inflammatory nature of UC leading to MM, as discussed in the previous paragraph.

The era of newer treatments in IBD, including biologics, has helped unveil a new mechanism through which IBD can be associated with MM. Liu et al. reported two cases of UC and MM associated with biologics. In the first case, the patient was diagnosed with MM shortly after the initiation of daclizumab. The patient’s UC remained silent after MM treatment. In the second case, the patient developed MM one year after the initiation of infliximab for the management of UC. The patient was found to have thrombocytopenia, anemia, and fatigue, and was eventually diagnosed with MM; however, given the delay of diagnosis, the patient passed away shortly after diagnosis [38]. Similar findings were reported by Yadav et al., where Infliximab use in a UC patient was associated with aggressive metastatic disease [5]. TNF-α inhibitors in IBD can lead to decreased apoptosis of plasma cells, thus setting the stage for the development of MM [7].

The results herein are important for several reasons. Clinicians should place a high degree of suspicion for MM in patients with UC, especially in the patient population that is historically known to be at risk for MM. UC patients with multiple cytopenias, including anemia, should be further evaluated as well [4]. Prolonged gastrointestinal symptoms in patients with known MM should raise clinical suspicion for possible UC [32].

Despite these robust findings, several limitations of our study must be acknowledged. First, the dataset used relies on administrative (ICD) codes, which carry the potential for misclassification. Second, while the accuracy of ICD-10 codes used herein has not been prospectively validated, we reviewed previously published manuscripts on the topic to confidently include the most appropriate codes [7,8]. Third, we were unable to access the impact of UC severity, phenotype, extra-intestinal manifestations, or its complications on MM prevalence. Fourth, because of the lack of granularity of this dataset, it remains unclear whether UC or immunosuppressive therapy, alone or in combination, have a significant role in precipitating MM. Finally, we acknowledge the fact that our study is not able to capture data in the outpatient setting, data which would provide a greater context, and/or perhaps a more accurate picture of the association between UC and MM. This could lead to Bergson’s bias. Furthermore, the dataset was mainly in the inpatient, making it difficult to extrapolate these results to the outpatient setting. These are somewhat attenuated by the relatively large US representative population included, which helps increase the internal validity.

## 5. Conclusions

In conclusion, by way of this large, nationally representative cross-sectional analysis, a significant association was identified between MM and UC. Moreover, we highlighted specific demographic subgroups of patients with UC that are at high risk of developing MM. Hence, a high degree of clinical suspicion of MM is warranted in patients with UC—especially in the demographic subgroups found to be at higher risk. In this era of precision medicine and increasing use of biologics for treatment of UC, future prospective data are needed to evaluate the impact of immunosuppressive therapy on the development of hematologic malignancies in patients with UC. Furthermore, we hope to encourage clinicians to keep MM in the differential diagnosis of UC patients with unexplained blood derangements, especially in the demographic subgroups at higher risk, as evidenced in this study. Further studies on the subject may lead to an update of guidelines that could implement systematic screening for MM in UC patients at higher risk.

## Figures and Tables

**Table 1:** The prevalence of MM among patients with and without UC between 2015–2018.

Characteristics	Prevalence of MM		
	Crude Prevalence		Multivariate Analysis
	*n*	%	
Patients with UC	1750	0.44	OR 1.26; (95% CI, 1.04–1.58) (*p* < 0.05)
Patients without UC	366,265	0.37	1 (reference)

Sample size: *n*, odds ratio: OR and confidence interval: CI.

**Table 2:** The prevalence of MM among UC patients stratified by subgroups between 2015–2018.

Characteristics	Prevalence of MM	
	Percentage (%)	95% CI
**Sex**		*p* = 0.03
Male patients with UC	53.7%	OR 1.36; (CI, 1.02–1.87)
Female patients with UC	46.3%	OR 1.14; (CI, 1.03–1.32)
**Ethnicity among patients with UC**		*p* = 0.001
Africa/American	15.6%	OR 1.31; (CI, 1.11–1.63)
White	9.2%	OR 1.05; (CI, 1.02–1.18)
**Age among patients with UC**		*p* < 0.001
≥65	70.9%	OR 2.7; (CI, 2.38–2.85)
<65	29.1%	1
**BMI**		*p* < 0.001
Obese (BMI > 30)	12.6%	OR 2.2; (CI, 2.17–2.61)
Non-obese	8.3%	1

OR: odds ratio; BMI: body mass index; CI: confidence interval.

## Data Availability

The data used and/or analyzed in this study are available in the National Inpatient Sample (NIS).
